# Immunological Characteristics of Patients Receiving Ultra-Short Treatment for Chronic Hepatitis C

**DOI:** 10.3389/fcimb.2022.885824

**Published:** 2022-06-27

**Authors:** Lone Wulff Madsen, Peer Brehm Christensen, Anne Øvrehus, Dorthe Marie Sjødahl Bryde, Dorte Kinggaard Holm, Søren Thue Lillevang, Christian Nielsen

**Affiliations:** ^1^ Department of Infectious Diseases, Odense University Hospital, Odense, Denmark; ^2^ Clinical Institute, University of Southern Denmark, Odense, Denmark; ^3^ Department of Clinical Immunology, Odense University Hospital, Odense, Denmark

**Keywords:** chronic hepatitis C, short treatment, PD-1, immune response, DAA, inhibitory receptor, SVR (sustained virologic response)

## Abstract

Reducing the treatment duration for chronic hepatitis C could be an important tool in the effort to reach the elimination goals set by the World Health Organization. The current challenge is to predict the target group who will achieve sustained virological response at week 12 (SVR12) with shorter treatment duration. The aim of this exploratory study was to characterize immune subsets with focus on inhibitory receptors in patients who experienced SVR12 or virological relapse following four weeks treatment with glecaprevir/pibrentasvir with or without ribavirin. A total of 32 patients were included in this study of whom 21 achieved SVR12 and 11 had virological relapse. All available samples at baseline (n = 31) and end of treatment (EOT) (n = 30) were processed for flow cytometric analysis in order to measure the expression of PD-1, 2B4, BY55, CTLA-4, TIM-3 and LAG-3 on 12 distinct T cell subsets. At baseline, patients with SVR12 (n=21) had numerically lower frequencies of inhibitory receptors for 83% (60/72) of the investigated T-cell subtypes. The most significant difference observed between the two groups was a lower frequency of stem cell-like memory T-cells CD4^+^PD1^+^ in the SVR group (p = 0.007). Furthermore, we observed a significant positive correlation between baseline viral load and the expression of PD-1 on the total CD8^+^ T-cells and effector memory T-cells CD4^+^ and CD8^+^ for patients with virological relapse. This study suggests a measurable immunologic phenotype at baseline of patients achieving SVR12 after short treatment compared to patients with virological relapse.

## Introduction

High cure rates of chronic hepatitis C virus (HCV) infection after treatment with direct acting antivirals (DAA) for 8-12 weeks has led to the speculation of possible reduced treatment duration. However, most studies with treatment duration for only 4 weeks have primarily achieved disappointing results (Jones et al., 2019; Cooke et al., 2021; Madsen et al., 2021). In order not to compromise high cure rates at the expense of shorter treatment duration, the current challenge is to identify the target population responsive to short-term treatment. Factors such as female gender, low baseline viral load, treatment naïve, genotype non 3, absence of baseline resistance-associated substitutions (RAS) and advance liver disease have all been shown to be predictors for achieving week 12 sustained virological response (SVR12) (Cavalcante and Lyra, 2015; Werner et al., 2016).

Increasing evidence has shown that host immunity also plays an important role in the response to DAA therapy (Rehermann and Thimme, 2019). Chronic infection with HCV is characterized by an unsuccessful endogenous interferon response and a failed response by virus specific CD8^+^ T cells mainly due to viral escape and T-cell exhaustion (Heim and Thimme, 2014). Prolonged and persistent HCV antigen exposure causes the exhaustion of T cells, a phenomenon which describes the progressive loss of effector functions and increased expression of inhibitory receptors (IR), which plays an important role in the regulation of the adaptive immune response (Wieland et al., 2017). Nonetheless, studies have indicated that the immune system could contribute to a DAA mediated HCV clearance. Higher baseline expression level of hepatic interferon stimulated genes (ISGs) is a predictor of SVR12 after DAA treatment (Alao et al., 2018) and HCV clearance by DAA therapy causes a downregulation of ISGs with a shift in the interferon response (Meissner et al., 2014) and a restoration of a normal natural killer (NK) cell phenotype and function (Serti et al., 2015; Nakamura et al., 2018). In addition, the HCV antigen removal mediated by DAA restores the function of HCV specific CD8^+^ T cells including decreased levels of PD-1 expression (Martin et al., 2014).

Current knowledge of the role of the immune system during short-term treatment with DAA is limited. Romani et al. showed higher levels of PD-1^+^ CD8^+^ T lymphocytes co-expressing either Tim-3, CD160, 2B4, KLRG1 or Blimp-1 at baseline and by end of treatment (EOT) in HCV patients who achieved SVR compared with those who relapsed (Romani et al., 2019). HCV specific CD8^+^ T cells with cytotoxic capacity were predominantly contained within these IR expressing PD-1^+^ subsets. Furthermore, pretreatment expression of specific markers (GZMB, PRF1, NKp46), associated with NK-cell and cytotoxic T cell function, have been associated with virological relapse after treatment with 4-6 weeks of DAA (Orr et al., 2021).

For a clinical biomarker that predict SVR to ultra-short treatment to be useful in clinical practice, the measurement should be simple and fast performed under routine conditions.

Therefore, the objective of our exploratory study was to measure and characterize simplified immune subsets with focus on inhibitory receptors to examine the potential for possible future clinical biomarkers for achieving SVR12 in patients who received four weeks treatment with glecaprevir/pibrentasvir (GLE/PIB) with or without ribavirin. In this study we chose to investigate some of the inhibitory receptors that previously have been investigated in relation to short treatment (PD-1, BY55, 2B4, CTLA-4, Tim-3 and LAG3).

## Materials and Methods

### Patients and Samples

A total of 32 treatment-naïve patients with chronic hepatitis C were enrolled in this study. The study was conducted at the Department of Infectious Diseases at Odense University Hospital in Denmark from May 2018 to August 2019. Originally the patients were randomized to (GLE/PIB) with or without ribavirin and a total of 17 patients received GLE/PIB and 15 patients were treated with GLE/PIB plus ribavirin for 4 weeks (Madsen et al., 2021). However, in the present study our goal was to compare baseline and EOT measures of inhibitory receptors for patients achieving SVR12 with nonSVR12 patients. Patients with all HCV genotypes were accepted and all included patients were < 50 years and had no severe liver disease and absence of fibrosis, defined as a liver stiffness measurement, measured by Fibroscan < 8Kpa. All patients with virological relapse were characterized as treatment failure. Blood samples utilized in this study were taken at baseline by treatment start and by EOT.

### Human Peripheral Blood Mononuclear Cell Isolation

Peripheral blood mononuclear cells (PBMCs) were isolated from whole blood using density gradient centrifugation (Lymphoprep™) and cryopreserved using DMSO-containing freezing medium and stored at – 196°C until flow cytometric analyses.

### Immunofluorescent Staining and Flow Cytometric Analysis

Multicolor flow cytometric analyses were performed on a BD FACSCanto™ II (BD Biosciences) and data were analyzed in FlowJo (TreeStar).

Concentration adjusted PBMCs were stained with the following backbone fluorochrome-conjugated monoclonal antibodies: anti-CD4 APC (clone SK3), anti-CD8 BV421 (clone RPA-T8), anti-CD45RO APC-H7 (clone UCHL1), anti-CCR7 PerCP-Cy5.5 (clone 150503), anti-CD95 PE-Cy7 (DX2). In separate tubes additional anti-PD1 PE (MiH4), anti-2B4 PE (2-69), anti-CTLA-4 PE (BNI3), anti-TIM-3 PE (7D3) from BD Biosciences or anti-LAG-3 PE (7H2C65) from BioLegend were added. All antibodies were titrated before use.

We used CD45RO, CCR7 and CD95 for dividing CD4^+^ and CD8^+^ in following T cell subsets (Mahnke, 2013) naïve T cells (Tn), stem cell-like memory T cells (Tscm), central memory T cells (Tcm), effector T cells (Te) and effector memory T cells (Tem). The subsets were identified as follows: CD45RO^-^CCR7^+^CD95^-^ Tn, CD45RO^-^CCR7^+^CD95^+^ Tscm, CD45RO^+^CCR7^+^ Tcm, CD45RO^-^CCR7^-^ Te, and CD45RO^+^CCR7^-^ Tem, see **Supplementary Figure 1**.

The T cell subsets were examined for the expression of the following inhibitory receptors: PD1 (CD279), 2B4 (CD244), BY55 (CD160), CTLA-4 (CD152), TIM-3 (CD366), and LAG-3 (CD223).

### HCV RNA

HCV RNA load was determined by the COBAS HCV assay (Roche Diagnostics GmbH, Mannheim, Germany) run at the cobas 6800 system (Roche Molecular system). The lower detection limit of the COBAS HCV assay was 15 IU/ml.

All analyses were performed according to the manufacturer’s instruction.

### Statistics

Descriptive statistics for continuous variables were reported as medians with interquartile ranges (IQR) for continuous variables. To test significance between groups we used *t* test for normally distributed data and Wilcoxon Mann-Whitney test for non-normally distributed data. Spearman’s rank correlation was used to describe the correlation between two variables. A p-value < 0.05 was considered significant. As the aim of the present study was exploratory, no adjustment for multiple testing was performed.

## Results

### Decreased Frequency of Inhibitory Receptors at Baseline for Patients Who Achieve SVR12 Compared to Patients With Virological Relapse

Overall, 31 samples were analyzed at baseline and 30 samples were analyzed at EOT. One patient from the ribavirin group who experienced virological relapse had missing sample at baseline and two ribavirin treated patients obtaining SVR12 had missing samples by EOT. **Table 1** provides baseline characteristics and treatment outcome after 4 weeks treatment. No significant association between SVR12 patients and baseline variables were identified.

**Table 1:** Baseline demographic and clinical characteristics of study patients according to SVR12 status after treatment.

Study population, n (%)	SVR12(n=21)	Virological relapse(n=11)
GLE/PIB + ribavirin	11	4
GLE/PIB	10	7
Age (years) median (range)	43 (28-49)	40 (34-48)
Male	14 (66.7)	9 (81.8)
BMI, median (IQR)	27.1 (25.9-33.2)	25.3 (21.7-27.5)
Past alcohol overuse Current alcohol overuse	11 (52.4) 1 (4.8)	7 (63.6) 0 (0)
Current or past intravenous drug use	15 (71.4)	9 (81.8)
HCV Genotype 1a/1b/2/3a/3b/4	7/0/2/11/0/1	4/2/2/1/1/0*
Baseline HCV RNA (log_10_ IU/ml) median (IQR)	6.47 (5.76-6.84)	6.60 (6.22-7.00)
Baseline ALT, median (IQR)	53 (41-70)	56 (42-107)
INFL 3 genotype CC/non CC	8/13	1/10
LSM in kPa, median (IQR)	5.6 (4.6-7.0)	4.7 (4.5-5.3)
Year since infected, median (IQR)	22 (10-27)	21 (18-26)

IQR, Interquartile range; BMI, Body Mass Index; HCV, Hepatitis C; ALT, alanine aminotransferase; LSM, Liver stiffness measurement.

*One patient with genotype 1 had a novel subgenotype (Pedersen et al., 2021).

**Figure 1:**
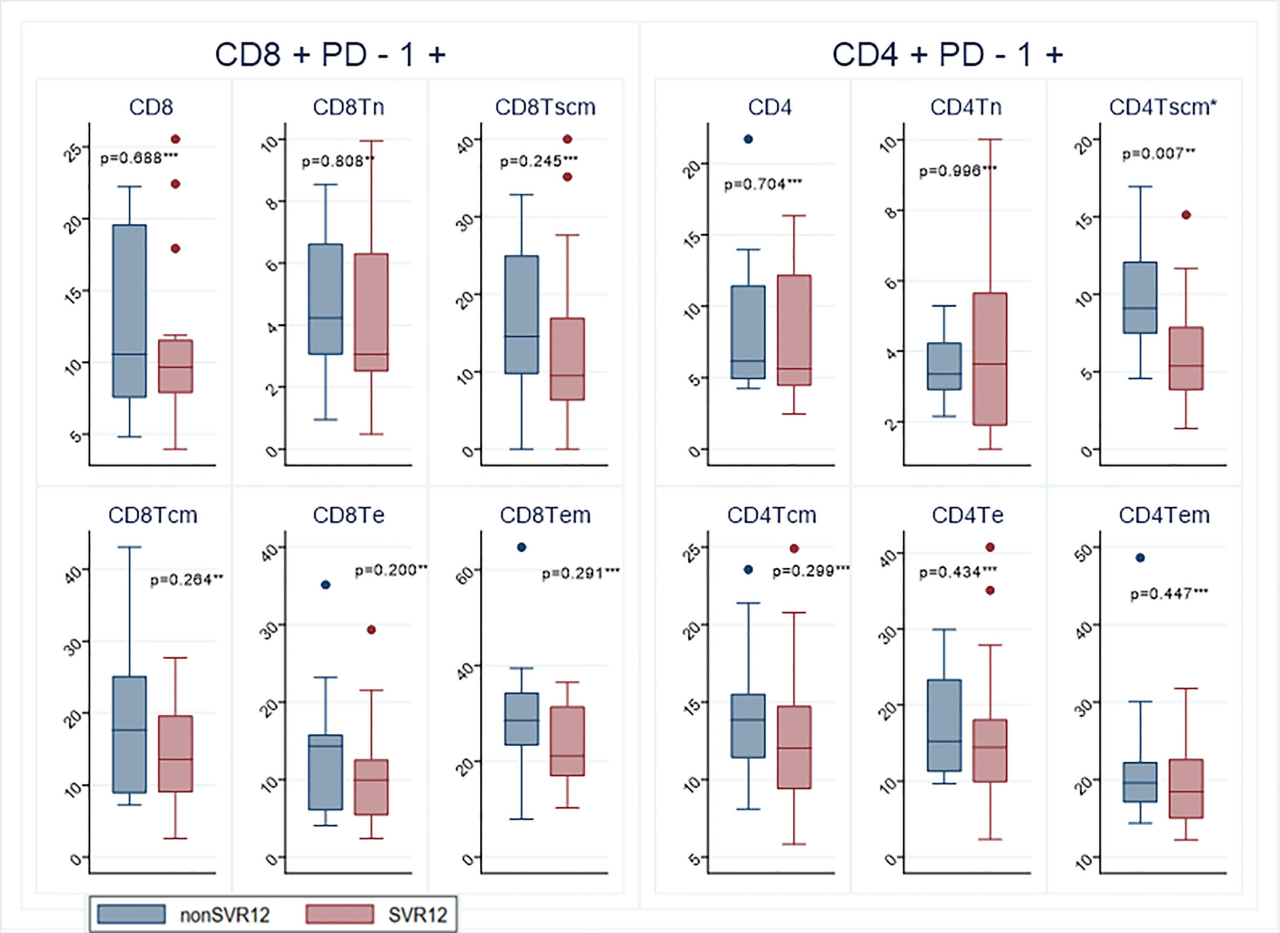
Expression of the inhibitory receptors PD1 on CD4+ and CD8+ lymphocytes at baseline from patients with virological relapse (non SVR) and patients who achieved cure (SVR). The colored dots represent measurements outside the IQR range. Sustained virological response (SVR); naïve T cells (Tn); T stem cells memory T-cells (Tscm); central memory T cells (Tcm); terminal effector T-cells (Te); effector memory T cells (Tem). Significant values marked with *. Statistical analyzes according to test for significance ** *t* test *** Wilcoxon Mann-Whitney test.

**Figure 2:**
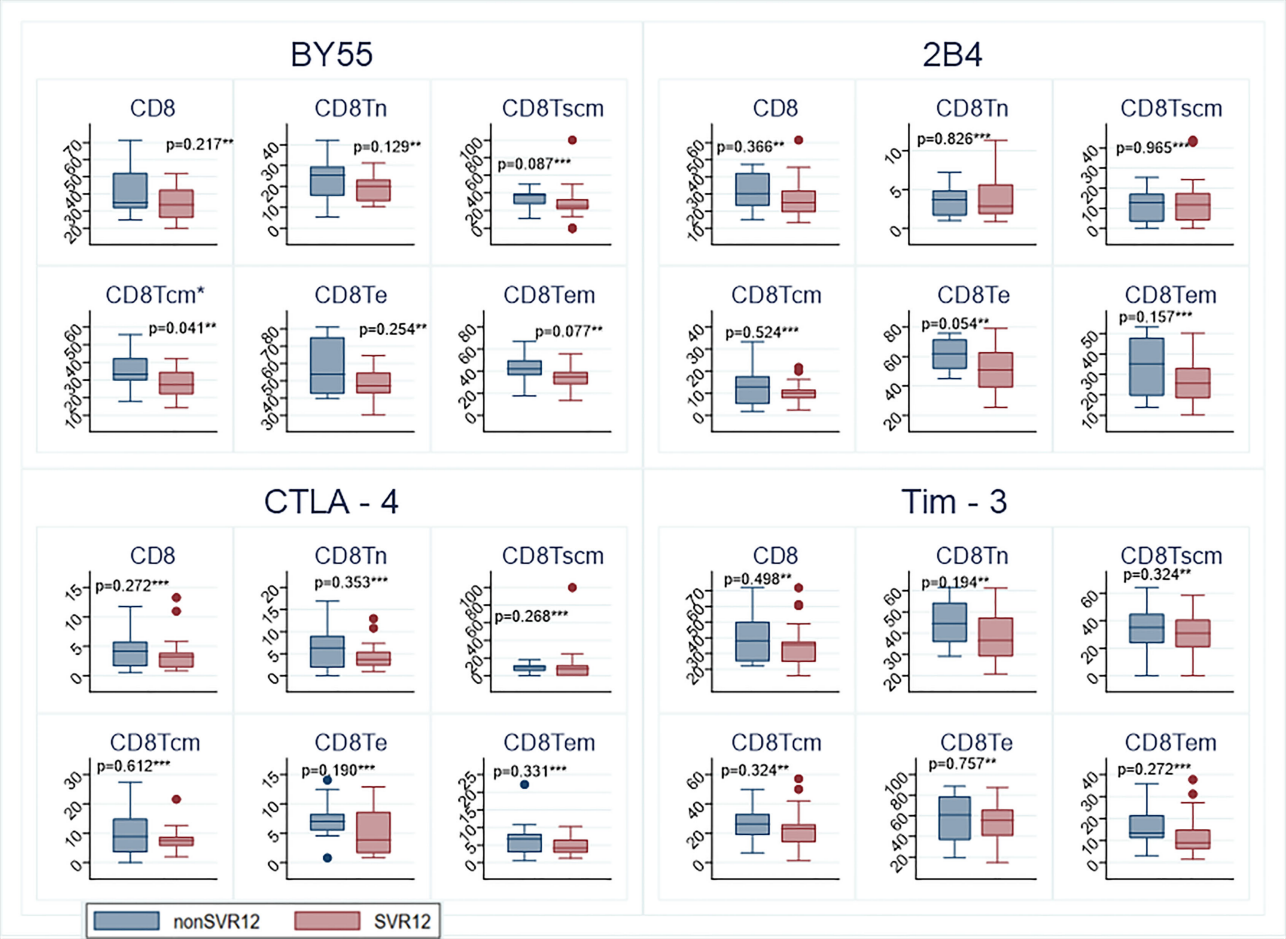
Expression of the inhibitory receptors BY55, 2B4, CTLA-4 and Tim-3 on CD8+ T cells at baseline from patients who achieved cure and patients with treatment failure. The colored dots represent measurements outside the IQR range. sustained virological response (SVR); naïve T cells (Tn); stem cell memory T-cells (Tscm); T central memory cell (Tcm); terminal effector T-cells (Te); T effector memory cell (Tem). Significant values marked with *. Statistical analyzes according to test for significance ** *t* test *** Wilcoxon Mann-Whitney test.

In general, patients who achieved SVR12 had lower frequencies of IRs at baseline. In total, 6 IRs were measured on 12 different T lymphocyte subtypes. Using the hypothesis-generating assumption that the overall expression of T-cell subtypes were not correlated and unrelated to SVR12 we would expect that for 50% of the subtypes SVR12 patients would had lower frequency. However, we observed that this was the case for 83.3% which is statistically higher than expected (p<0.001).This was most pronounced for BY55 and Tim3 where all SVR12 patients had lower median level for all T-lymphocytes as well as for PD-1 and CTLA-4, where the estimated median level was numerically lower for 11/12 measured subtypes, see **Supplemental 2**. The most statistically significant difference observed between the two groups was for stem cell memory T-cells CD4^+^PD1^+^ (p = 0.007) with the lowest frequency for the SVR12 patients (**Figure 1**). The estimated median frequency of CD8^+^ cells expressing either PD-1, BY55, 2B4, CTLA-4 or Tim-3 were lower for SVR12 patients than for patients with virological relapse (**Figures 1**, **2**). There was a statistically significant higher frequency of effector memory CD4^+^2B4^+^ T-cells among SVR12 patients than in patients with treatment failure.

By end of treatment, there was no significant difference for any markers between the two groups. The tendency for both groups were decreased frequencies of inhibitory receptors after treatment. Frequency of effector memory CD8^+^ T-cells decreased during treatment for the group who achieved SVR12 whereas an increase during treatment was observed for the group who experienced virological relapse (p=0.005).

### Positive Correlation Between PD-1 Expression on Distinct Subtypes of T-Cells and Baseline Viral Load for Patients With Virological Relapse

**Figure 3:**
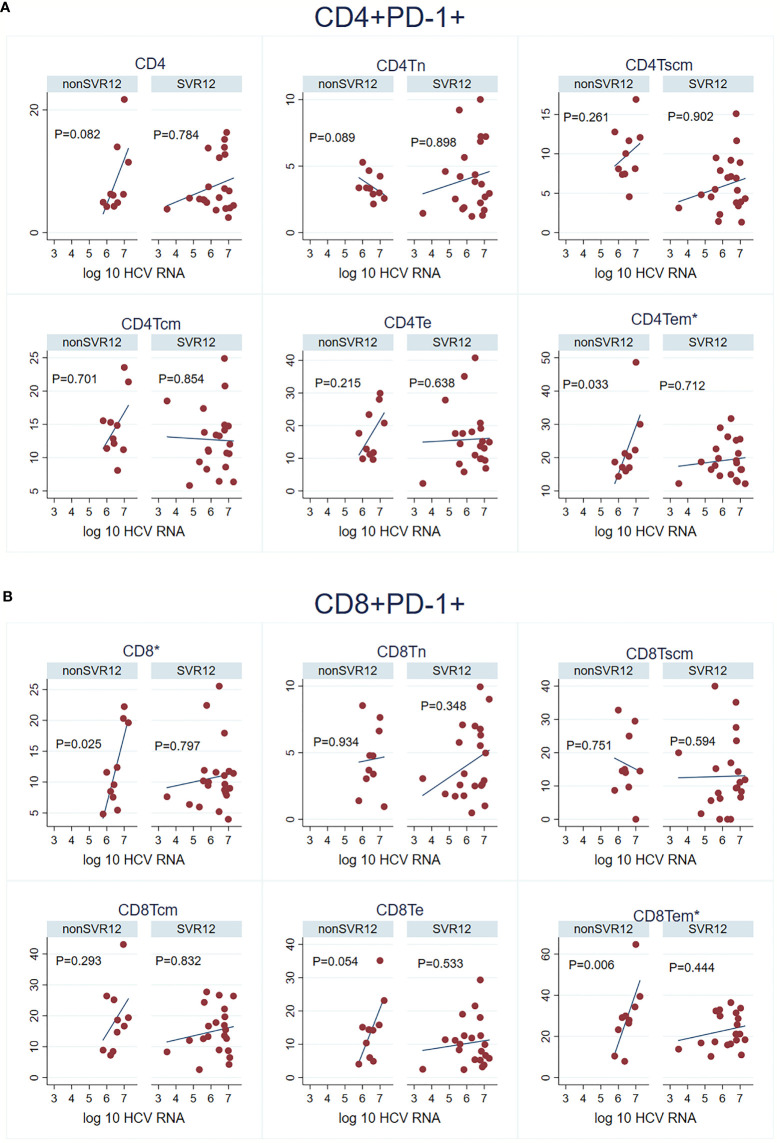
Viral load and the expression of PD-1 by baseline for the different subset of T-cells divided for patients who achieved SVR12 and virological relapse. **(A)** CD4^+^ PD-1^+^ and HCV RNA. **(B)** CD8^+^ PD-1^+^ and HCV RNA. Sustained virological response (SVR); naïve T cells (Tn); stem cell memory T-cells (Tscm); T central memory cell (Tcm); terminal effector T-cells (Te); T effector memory cell (Tem). Significant values marked with *.

For clinical parameters, we investigated whether there was any correlation between viral load and the expression of PD-1. In general, patients who experienced virological relapse had a tendency to increasing expression of PD-1 with higher viral load. This positive correlation was found significant for PD-1 expressing CD4^+^ and CD8^+^ effector memory cells (Spearman’s rho = 0.673, p=0.033 and rho = 0.794, p=0.006, respectively. In addition, high level of CD8^+^ cells had a significant correlation with high viral load (rho = 0.697, p=0.025 for the group with virological relapse (**Figures 3A, B**).

## Discussion

This study shows that patients who achieved SVR12 after 4 weeks treatment with DAA for chronic hepatitis C had an overall lower expression of IR’s compared to patients who experienced virological relapse. This indicates that the T-cells of patients with SVR12 has a less activated and anergic phenotype at baseline compared to the relapse group, correlating with better treatment outcome. The estimated median frequency of all CD8^+^ cells expressing either PD-1, BY55, 2B4, CTLA-4 or Tim-3 were lower for SVR12 patients than for patients with virological relapse. These findings are contrary to the study of Romani et al. who found higher frequencies of HCV specific CD8^+^ T lymphocytes co-expressing PD-1 with other IRs in patients who achieved SVR12 after 4 weeks treatment with DAA (Romani et al., 2019). There are several differences between our study and the study by Romani et al, which limit the comparability. In our study, we investigated the total population of T-lymphocytes and did not study HCV specific T-lymphocytes and secondly we did not investigate the co-expression of IRs. Both studies imply that the largest difference in T-cell characteristics in SVR12 patients compared to those with virological relapse lies in the distribution of the CD8^+^ T- cells population. Studies indicate that HCV specific CD8^+^ cells are the major antiviral effector cells and are maintained during persistent infection while the HCV specific CD4^+^ cells mainly have a regulatory function with a rapid decline and deletion after acute infection (Schulze Zur Wiesch et al., 2012; Kemming et al., 2020). The lack of CD4^+^ T cell help is one of the hallmarks of the T cell exhaustion during chronic infection. In our study we found that the most significant marker between the two groups was stem cell memory T-cells CD4^+^PD1^+^ with the lowest level for the SVR group at baseline (p = 0.007).

In this study we did not find any significant difference for inhibitory receptors for SVR12 and non SVR12 patients by EOT. However, this must be interpreted with caution as confounding by ribavirin treatment in some patients could produce biased results. One of the possible modes of action by ribavirin is through an immunomodulatory host response to a swift to a more antiviral Th1 profile (Tam et al., 1999; Langhans et al., 2012) and therefore it may also influence the expression of inhibitory receptors. However as the differences observed in this study was at baseline before ribavirin was given we do not think it was an important confounder in this study.

The hypothesis of DAA mediated immune restoration is that antigen removal leads to the reconstitution of the peripheral T-cell population. In this study, all patients except one with virological relapse had no measurable virus by EOT. This is reflected by the fact that the majority of the IRs decreased in frequency for both groups during treatment. Effector memory CD8^+^ T-cells decreased during treatment for the group who achieved SVR12 whereas we observed an increase during treatment for the group who experienced virological relapse (p=0.005). Whether this is due to lack of immune activation for the group with virological relapse is speculative. However, one patient in this study achieved SVR12 despite of positive viral load by post treatment week 4, which is likely to be the result of immune driven elimination of residual viral replication (Maasoumy et al., 2018). We have previously showed for the same cohort that patients with SVR12 had higher levels of distinct soluble inflammatory mediators by EOT but not at baseline which support the theory that a certain immune activation is needed to control residual viremia if DAA is terminated early (Khera et al., 2022).

The polymorphisms in the interferon lambda 3 (INFL3) gene encoding interferon λ-3 has been strongly associated with spontaneously HCV clearance and treatment response to pegylated interferon and ribavirin (Hayes et al., 2012). The role of the INFL3 genotypes in the DAA era is less important in relation to treatment outcome but the favorable genotype CC has been described with better treatment response after short treatment (Lawitz et al., 2017). In our study there was no significant association between SVR12 and genotype CC. However, only one patient with genotype CC failed 4 weeks treatment and this patient also had double baseline NS5A resistance-associated substitutions (30K, 31M) (Madsen et al., 2021).

The functionally impaired function of the HCV specific CD8^+^ cells in chronic hepatitis C is characterized by an upregulation of inhibitory receptors. In this study, we found that the inhibitory receptors BY55, Tim3, PD -1 and CTLA-4 had higher expression level at baseline for patients with virological relapse. The level of PD-1 expression has been shown to be directly correlated to functional impairment of the HCV specific CD8^+^ T-cells (Nakamoto et al., 2008). PD-1 blocking therapy is already an established treatment in cancer therapy but the role of PD-1 blocking therapy in chronic infection is less defined (Jubel et al., 2020). A randomized study showed that anti-PD-1 monoclonal antibody can reduce HCV RNA and that a previously null responder of interferon therapy achieved SVR12 after PD-1 blocking treatment (Gardiner et al., 2013). However, the level and pattern of co-expression of IRs seems to be crucial for the T-cell exhaustion as simultaneous blocking of IRs can synergistically improve T cell response and diminish viral load (Blackburn et al., 2009).

In our study, we found a positive correlation between baseline viral load and high expression frequency of PD-1 on total CD8^+^ cells (Spearman’s rho = 0.697, p=0.025) and on effector memory T-cells CD8+ (rho= 0.7939 p= 0.0061) and CD4+ (rho = 0.673, p= 0.033) for patients with treatment failure. The same positive correlation has been found for both untreated patients with HIV infection and for chronic hepatitis C (Day et al., 2006; Shen et al., 2010). This indicates that increasing amount of antigen is associated with increased expression of PD-1 on distinct types of T-cells. Interestingly, we only found this positive correlation for patients with virological relapse and future clinical validations need to clarify whether this is a useful predictive biomarker. High expression of PD-1 has previously been correlated to advanced liver fibrosis (Osuch et al., 2020) and furthermore high pretreatment expression of PD-1 has been found to be negatively associated with SVR for African Americans but not for Caucasians (Golden-Mason et al., 2008). Both factors associated with lower treatment response to interferon and ribavirin (Conjeevaram et al., 2006).

The major strength of our study is that included patients have the favorable clinical characteristic to be curable with shorter treatment duration and this patients group belongs to the largest untreated group in many high-income countries.

The major limitations of the present study is that the results are based on a reuse of data collected in a randomized controlled trial study for treatment effect of ribavirin. The small sample size does not allow for statistical adjustment for potential confounding factors. Hence, results should be interpreted with caution. Secondly, it is a limitation for this study that we only investigated the total T-cell population and not the co-expression of IRs on HCV specific T-cells. However, if immunological markers should be useful in clinical decision-making for treatment duration, their measurement should be rapid and relatively easily performed. Many factors are known to have influence on the immune system herein alcohol and illegal drug use which was reported frequently in this cohort. The small sample size means that it has not been possible to take these confounders into account. Therefore the described findings with a potential for a clinical marker needs to be confirmed by larger studies. PD-1 correlated to viral load could be an area that would be interesting to investigate further not only as a potential marker but also in the aspect to better understanding of the interaction between virus and host immune response.

## Conclusion

This exploratory study showed lower expression frequency of IRs on T-cells at baseline for patients who later achieved SVR12 after ultrashort treatment for chronic hepatitis C. Furthermore, we found a positive correlation between the expression of PD-1 on distinct subtypes of T-cells and viral load exclusively for patients with virological relapse.

## Data Availability Statement

Due to Danish rules on data availability, we are unable to make an anonymized dataset public. These rules are based on the Data Protection Act, imposed by The Danish Data Protection Agency. An English translation of the Data Protection Act can be found on the official website for The Danish Data Protection Agency (https://www.datatilsynet.dk/english/legislation/). For further information, the corresponding author can be contacted. Requests to access the datasets should be directed to lone.wulff.madsen@rsyd.dk.

## Ethics Statement

This study was reviewed and approved by Danish Health and Medicines Authorities, Eudra CT no: 2017-005179, The Regional Committees on Health Research Ethics for Southern Denmark ID-S20180013 and the Danish Data Protection agency (j.no 18/ 21965). All patients signed informed written consent for the study. All patients who experienced virological relapse were offered retreatment of whom 10 patients have been retreated and all achieved SVR12.

## Author Contributions

AØ and PC designed the clinical study and AØ, PC, and LM were involved in patient recruitment. The immunological study was conceived by CN, SL, DH, and DB with input from all authors. CN, SL, and DH performed and analyzed the immunological analyses. LM conducted the data analyses and drafted the manuscript with input from all authors. All authors have seen and approved the final manuscript.

## Funding

Financial support was given from a public fund from the Danish Regions Medicines Research fund EMN-2017-00901 and a Ph.D. fund from the Region of Southern Denmark and the University of Southern Denmark, neither University of Southern Denmark, neither of whom had any role in any part of this study.

## Conflict of Interest

PC and AØ have received research grants from AbbVie, Gilead and MSD, not related to this study and have received travel and conference support from AbbVie, Gilead and MSD.

The remaining authors declare that the research was conducted in the absence of any commercial or financial relationships that could be construed as a potential conflict of interest.

## Publisher’s Note

All claims expressed in this article are solely those of the authors and do not necessarily represent those of their affiliated organizations, or those of the publisher, the editors and the reviewers. Any product that may be evaluated in this article, or claim that may be made by its manufacturer, is not guaranteed or endorsed by the publisher.
