# Supplementary material for: Immunological Characteristics of Patients Receiving Ultra-Short Treatment for Chronic Hepatitis C
Source: Front Cell Infect Microbiol. 2022 Jun 27;12:885824. doi: 10.3389/fcimb.2022.885824 (PMC9271618; doi:10.3389/fcimb.2022.885824)
Supplement: Supplementary file 1 [file DataSheet_1.docx]

Supplementary Material

Supplementary 1

Gating strategy for identifying the frequency of and expression of different inhibitory receptors on Tem, Tcm , Te, Tn-like, Tn and Tscm CD4 and CD8 T cell subsets.


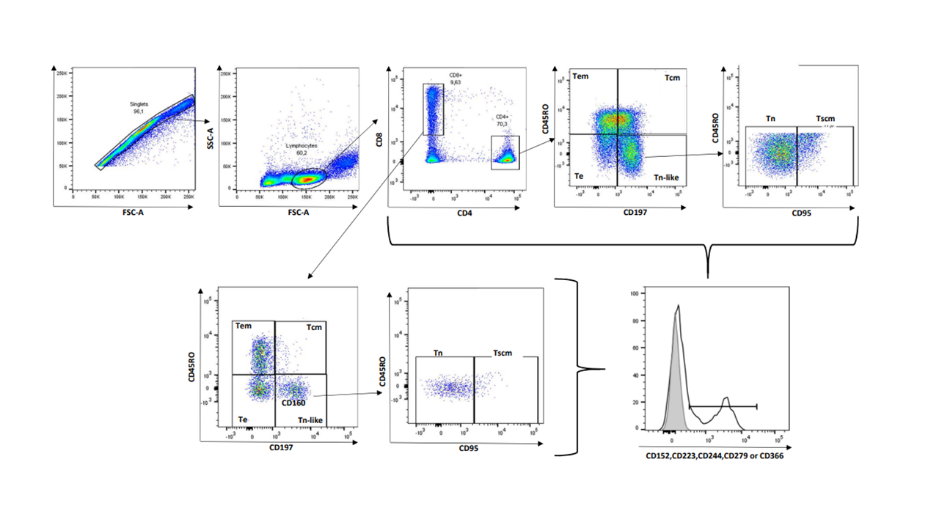


Abbreviations: naïve T cells (Tn); T stem cell memory (Tscm); T central memory cell (Tcm); terminal effector T-cells (Te); T effector memory cell (Tem);

Supplementary 2

Tabel S2 – Median values for the different cell types for the SVR12 and non-SVR12 group at baseline. Significant values marked with *

| % of parent population | **Parent population** | **SVR (21)**  **Median (IQR)** | **Non SVR (10)**  **Median (IQR)** | **P-values** |
| --- | --- | --- | --- | --- |
| CD4+ | Lymphocytes | 54.36 (47.43 -59.00) | 55.54 (45.88 -59.16) | 0.6728 |
| CD8+ | Lymphocytes | 13.83 (11.34-16.4) | 14.51 (12.72-18.08) | 0.3525 |
| CD4+Tcm | CD4+ | 18.34 (9.04-25.38) | 15.10 (12.20 - 21.03) | 0.8877 |
| CD4+Te | CD4+ | 9.61 (6.63-15.13) | 7.46 (5.98-21.15) | 0.4725 |
| CD4+Tem | CD4+ | 19.98 (14.74-25.14) | 16.49 (13.12-20.38) | 0.2265 |
| CD4+Tn-like | CD4+ | 46.76 ( 34.54-52.39) | 55.77 (50.49-61.25) | 0.1139 |
| CD4+Tscm | CD4^+^CD45RO^-^CD197^+^ | 9.74 (6.33-13.48) | 12.82 (9.38-17.33) | 0.2125 |
| CD4+Tn | CD4^+^CD45RO^-^CD197^+^ | 90.30 (86.11-93.32) | 87.24 (82.40-90.35) | 0.2367 |
| CD8+Tn-like | CD8+ | 23.39 (16.03-28.85) | 19.32 (18.53-33.10) | 0.5541 |
| CD8+Tcm | CD8+ | 3.88 (2.97-4.99) | 3.64 (2.09-8.96) | 0.8658 |
| CD8+Te | CD8+ | 40.16 (33.77-49.06) | 49.27 (30.63-54.00) | 0.7998 |
| CD8+Tem | CD8+ | 30.92 (21.67-34.99) | 23.80 ( 17.34-29.46) | 0.1305 |
| CD8+Tscm | CD8^+^CD45RO^-^CD197^+^ | 8.49 (2.46-15.92) | 6.09 (4.59-11.75) | 0.8327 |
| CD8+Tn | CD8^+^CD45RO^-^CD197^+^ | 91.54 ( 84.21-97.55) | 93.93 (88.28-95.27) | 0.8658 |
| **PD1 (CD279)** |  |  |  |  |
| CD4+ | CD4+ | 5.62 (4.42-12.21) | 6.17 (4.88-11.47) | 0.7037 |
| CD4+Tn | CD4+ | 3.64 (1.89-5.66) | 3.35 (2.9-4.24) | 0.9663 |
| CD4+Tscm | CD4+ | 5.37 ( 3.8- 7.88) | 9.08 (7.45-12.09) | 0.0074* |
| CD4+Tcm | CD4+ | 12.02 (9.38-14.75) | 13.85 (11.39-15.55) | 0.2990 |
| CD4+Te | CD4^+^CD45RO^-^CD197^+^ | 14.45 (9.85-18.12) | 15.26 (11.21-23.4 ) | 0.4341 |
| CD4+Tem | CD4^+^CD45RO^-^CD197^+^ | 18.43 (14.95-22.64) | 19.56 (17.03-22.29) | 0.4469 |
| CD8+ | CD8+ | 9.66 (7.87-11.58) | 10.58 (7.55-19.62) | 0.6881 |
| CD8+Tn | CD8+ | 3.06 (2.5-6.32) | 4.23 (3.05-6.63) | 0.8075 |
| CD8+Tscm | CD8+ | 9.47 (6.25-16.95) | 14.51 (9.68-25) | 0.2447 |
| CD8+Tcm | CD8+ | 13.59 (8.97-19.68) | 17.66 (8.89-25.19) | 0.2641 |
| CD8+TeCD279 | CD8^+^CD45RO^-^CD197^+^ | 9.93 (5.38-12.59) | 14.33 (6.01-15.81) | 0.2000 |
| CD8+TemCD279 | CD8^+^CD45RO^-^CD197^+^ | 21.16 (16.84-31.44) | 28.53 (23.27-34.36) | 0.2908 |
| **BY55 (CD160)** |  |  |  |  |
| CD4+ | CD4+ | 14.43 ( 9.96-17.36) | 16.47 ( 12.67-22.77) | 0.2549 |
| CD4+Tn | CD4+ | 16.72 (13.56-19.72) | 23.94 (17.63-26.57) | 0.0501 |
| CD4+Tscm | CD4+ | 15.1 (11.57-21.3) | 19.56 (16.22-27.09) | 0.0861 |
| CD4+Tcm | CD4+ | 19.02 (13.05-24.19) | 23.97 (17.58-29.06) | 0.2203 |
| CD4+Te | CD4^+^CD45RO^-^CD197^+^ | 20.26 (16.96-29.14) | 26.04 (19.13-30) | 0.4966 |
| CD4+Tem | CD4^+^CD45RO^-^CD197^+^ | 17.85 (14.69-24.29) | 21.41 (18.03-25.2) | 0.3345 |
| CD8+ | CD8+ | 33.72 (25.97-42.54) | 35.04 (31.61-52.35) | 0.2169 |
| CD8+Tn | CD8+ | 20 (12.97-23.39) | 25.48 (15.57-29.61) | 0.1294 |
| CD8+Tscm | CD8+ | 25 (21.31-33.33) | 37.75 (27.45-39.58) | 0.0866 |
| CD8+Tcm | CD8+ | 27.27 (21.9-34.74) | 33.33 (29.73-42.45) | 0.0416* |
| CD8+Te | CD8^+^CD45RO^-^CD197^+^ | 47.14 (42.62-54.83) | 53.82 (42.39-75.34) | 0.2539 |
| CD8+Tem | CD8^+^CD45RO^-^CD197^+^ | 35.06 (28.04-39.6) | 42.11 (36.32-50.05) | 0.0771 |
| **2B4 (CD244)** |  |  |  |  |
| CD4+ | CD4+ | 1.97 (1.04-2.93) | 1.5 (.45-2.52) | 0.3116 |
| CD4+Tn | CD4+ | 2.42 (1.61-3.38)  (n=20) | 2.21 (1.3-2.47) | 0.3909 |
| CD4+Tscm | CD4+ | 2.08 (1.48-2.74) | 1.58 (1.22-2.36) | 0.3787 |
| CD4+Tcm | CD4+ | 3.09 (2.14-6.4)  (n=20) | 3.31 (1.8-3.83) | 0.4545 |
| CD4+Te | CD4^+^CD45RO^-^CD197^+^ | 5.6 (2.52-9.19)  (n=20) | 2.71 (1.88- 8.93) | 0.4815 |
| CD4+Tem | CD4^+^CD45RO^-^CD197^+^ | 3.83 (2.45-5.79)  (n=20) | 2.13 (1.63-3.35) | 0.0366* |
| CD8+ | CD8+ | 25.09 (19.24-32.17)  (n=20) | 30.32 (23.18-42.38) | 0.3662 |
| CD8+Tn | CD8+ | 2.84 (1.81-5.68)  (n=20) | 3.68 (1.64-4.89) | 0.8259 |
| CD8+Tscm | CD8+ | 11.66 ( 3.87-17.71)  (n=20) | 12.87 (3.33-17.24) | 0.9649 |
| CD8+Tcm | CD8+ | 10.04 (7.62-11.72)  (n=20) | 12.92 (5.12-17.74) | 0.5235 |
| CD8+Te | CD8^+^CD45RO^-^CD197^+^ | 50.87 (38.77-62.97)  (n=20) | 61.76 (51.69-71.7) | 0.0543 |
| CD8+Tem | CD8^+^CD45RO^-^CD197^+^ | 25.67 (18.28-33.3)  (n=20) | 35.16 (19.54-48.07) | 0.1571 |
| **CTLA-4 (CD152)** |  |  |  |  |
| CD4+ | CD4+ | 2.72 (1.23-4.13) | 2.93 (2.04-4.48) | 0.7484 |
| CD4+Tn | CD4+ | 3.85 (2.46-5.56) | 4.87 (3.61-7.2) | 0.2204 |
| CD4+Tscm | CD4+ | 3.34 (1.78-5.34) | 4.66 (2.84- 8.08) | 0.1624 |
| CD4+Tcm | CD4+ | 5.71 (3.2-7.51) | 5.01 (4.8-7.85) | 0.6073 |
| CD4+Te | CD4^+^CD45RO^-^CD197^+^ | 6.02 (2.6-11.92) | 6.11 (2.78-9.63) | 0.8327 |
| CD4+Tem | CD4^+^CD45RO^-^CD197^+^ | 4.14 (2.93-6.23) | 4.92 (2.72-6.72) | 0.5262 |
| CD8+ | CD8+ | 3.27 (1.43-3.94) | 4.18 (1.57-5.83) | 0.2719 |
| CD8+Tn | CD8+ | 3.68 (2.32-5.45) | 6.26 (1.83-9.08) | 0.3525 |
| CD8+Tscm | CD8+ | 7.61 (0-12) | 10.44 (5.36-11.43) | 0.2678 |
| CD8+Tcm | CD8+ | 7.52 (5.71-8.94) | 8.90 (3.45-14.97) | 0.6121 |
| CD8+Te | CD8^+^CD45RO^-^CD197^+^ | 3.92 (1.63-8.68) | 7.04 (5.48-8.33) | 0.1902 |
| CD8+Tem | CD8^+^CD45RO^-^CD197^+^ | 4.15 (2.79-6.55) | 6.74 (2.89-8.2) | 0.3311 |
| **Tim-3 (CD366)** |  |  |  |  |
| CD4+ | CD4+ | 18.96 (14.36-25.03) | 20.94 ( 17.05-30.07) | 0.2203 |
| CD4+Tn | CD4+ | 32.45 (22.85-44.62) | 45.93 (30-49.87) | 0.3105 |
| CD4+Tscm | CD4+ | 15.6 (10.66-18.4) | 17.96 (14.97-25.13) | 0.1995 |
| CD4+Tcm | CD4+ | 13.27 (9.08-18.91) | 14.89 (8.91-20.29) | 0.7468 |
| CD4+Te | CD4^+^CD45RO^-^CD197^+^ | 19.25 (14.83-21.65) | 23.21 (18.29-33.33) | 0.2902 |
| CD4+Tem | CD4^+^CD45RO^-^CD197^+^ | 11.43 (7.11-14.12) | 13.64 (11.12-18) | 0.1083 |
| CD8+ | CD8+ | 35.65 (24.7-37.79) | 38.06 (25.05-50.34) | 0.4978 |
| CD8+Tn | CD8+ | 36.53 (29.02-47.42) | 44.46 (35.75-54.4) | 0.1938 |
| CD8+Tscm | CD8+ | 30.83 (20.61-40.88)  (n=20) | 35.05 (23.61-45) | 0.4658 |
| CD8+Tcm | CD8+ | 23.26 (13.89-26.13) | 26.44 (18.65-33.33) | 0.3242 |
| CD8+Te | CD8^+^CD45RO^-^CD197^+^ | 55.49 (40.49-66.01) | 60.74 (36.51-79.02) | 0.7565 |
| CD8+Tem | CD8^+^CD45RO^-^CD197^+^ | 9.12 (6.11-15.12) | 13.58 (11.11 -21.69) | 0.2719 |
| **LAG3 (CD223)** |  |  |  |  |
| CD4+CD223 | CD4+ | 1.5 (.77-2.15) | 1.50 (.92-3.45) | 0.7037 |
| CD4+TnCD223 | CD4+ | 2.63 (1.22-4.13) | 3.51 (2.35-4.32) | 0.3980 |
| CD4TscmCD223 | CD4+ | 2.12 (.81-3.4) | 2.35 (1.48-5.4) | 0.4219 |
| CD4+TcmCD223 | CD4+ | 4 (1.92-5.57) | 3.35 (2.34-4.6) | 0.7353 |
| CD4+TeCD223 | CD4^+^CD45RO^-^CD197^+^ | 4.18 (2.99-6.29) | 4.10 (1.53-8.39) | 0.9663 |
| CD4+TemCD223 | CD4^+^CD45RO^-^CD197^+^ | 2.61 (1.36-4.67) | 2.71 (1.36-4.23) | 0.9159 |
| CD8+CD223 | CD8+ | 2.29 (1.44-4.14) | 3.44 (1.74-3.97) | 0.6572 |
| CD8+TnCD223 | CD8+ | 2.95 (2-4.4) | 3.43 (2.14-4.07) | 0.5827 |
| CD8+TscmCD223 | CD8+ | 3.33 (1.69-8.7) | 3.45 (.97-10.53) (n=9) | 1.0000 |
| CD8+TcmCD223 | CD8+ | 5.81 (3.9-8.82) | 4.66 (2.27-8.53) | 0.5682 |
| CD8+TeCD223 | CD8^+^CD45RO^-^CD197^+^ | 3.84 (2.53-5.28) | 5.21 (3.48-9.51) | 0.1831 |
| CD8+TemCD223 | CD8^+^CD45RO^-^CD197^+^ | 3.2 (2.17-4.88) | 3.16 (2.31-5.76) | 0.6121 |

Abbreviations: sustained virological response (SVR); naïve T cells (Tn); T stem cell memory (Tscm); T central memory cell (Tcm); terminal effector T-cells (Te); T effector memory cell (Tem); Programmed cell death protein 1 (PD-1); cytotoxic T-lymphocyte-associated protein 4 (CTLA-4); T cell immunoglobulin and mucin domain-3 (Tim-3); Lymphocyte-activation gene 3 (LAG3).
